# A Novel Nine-lncRNA Risk Signature Correlates With Immunotherapy in Hepatocellular Carcinoma

**DOI:** 10.3389/fonc.2021.706915

**Published:** 2021-09-15

**Authors:** Ye Nie, Jianhui Li, Wenlong Wu, Dongnan Guo, Xinjun Lei, Tianchen Zhang, Yanfang Wang, Zhenzhen Mao, Xuan Zhang, Wenjie Song

**Affiliations:** ^1^Xi’an Medical University, Xi’an, China; ^2^Department of Hepatobiliary Surgery, Xijing Hospital, The Fourth Military Medical University, Xi’an, China; ^3^School of Pharmacy, Health Science Center, Xi’an Jiaotong University, Xi’an, China

**Keywords:** hepatocellular carcinoma, prognosis, tumor-infiltrating lymphocytes, immunotherapy response, tertiary lymphoid structure, ferroptosis

## Abstract

**Background:**

Hepatocellular carcinoma is one of the most common malignant tumors with a very high mortality rate. The emergence of immunotherapy has brought hope for the cure of hepatocellular carcinoma. Only a small number of patients respond to immune checkpoint inhibitors, and ferroptosis and tertiary lymphoid structure contribute to the increased response rate of immune checkpoint inhibitors; thus, we first need to identify those who are sensitive to immunotherapy and then develop different methods to improve sensitivity for different groups.

**Methods:**

The sequencing data of hepatocellular carcinoma from The Cancer Genome Atlas and Gene Expression Omnibus was downloaded to identify the immune-related long non-coding RNAs (lncRNAs). LncRNAs related to survival data were screened out, and a risk signature was established using Cox proportional hazard regression model. R software was used to calculate the riskScore of each patient, and the patients were divided into high- and low-risk groups. The prognostic value of riskScore and its application in clinical chemotherapeutic drugs were confirmed. The relationship between riskScore and immune checkpoint genes, ferroptosis genes, and genes related to tertiary lymphoid structure formation was analyzed by Spearman method. TIMER, CIBERSORT, ssGSEA, and ImmuCellAI were used to evaluate the relative number of lymphocytes in tumor. The Wilcoxon signed-rank test confirmed differences in immunophenoscore between the high- and low-risk groups.

**Results:**

Data analysis revealed that our signature could well predict the 1-, 2-, 3-, and 5-year survival rates of hepatocellular carcinoma and to predict susceptible populations with Sorafenib. The risk signature were significantly correlated with immune checkpoint genes, ferroptosis genes, and tertiary lymphoid structure-forming genes, and predicted tumor-infiltrating lymphocyte status. There was a significant difference in IPS scores between the low-risk group and the high-risk group, while the low-risk group had higher scores.

**Conclusion:**

The riskScore obtained from an immune-related lncRNA signature could successfully predict the survival time and reflect the efficacy of immune checkpoint inhibitors. More importantly, it is possible to select different treatments for different hepatocellular carcinoma patients that increase the response rate of immune checkpoint inhibitors and will help improve the individualized treatment of hepatocellular carcinoma.

## Introduction

Hepatocellular carcinoma (HCC) ranks the fourth in mortality among malignant tumors (1). Viral infection, aflatoxin exposure, smoking, and heavy alcohol consumption are common pathogenic factors leading to HCC (2). Areas including North and West part of Africa and East Southeast part of Asia have a high incidence of HCCs (3, 4). In China, the 5-year survival rate is <14.1% (5). The late onset of symptoms of HCCs makes early diagnosis difficult, and at the time of diagnosis, only one-third of patients are eligible for radical excision (6). The high recurrence rate and short survival time even after surgery indicate the urgent need for the development of precise individualized HCC treatment (7). Compared with traditional treatment, the application of immune checkpoint inhibitors (ICI) (anti-PD1, anti-PD-L1, and anti-CTLA4) has shown better prognostic value in the treatment of several tumors (8–10). At present, nivolumab and pembrolizumab have been used in the clinical treatment of HCC (11). In many cancers therapies, immunotherapy has become a hotspot treatment and is also the most likely treatment plan to achieve long-term survival. The main characteristics of immunotherapy are activation of tumor-infiltrating lymphocytes (TILs), killing of tumor cells, and inhibition of tumor metastasis and recurrence. However, a disadvantage of immunotherapy is that only a few people respond to ICI treatment. Therefore, research on the tumor microenvironment (TME) and tertiary lymphoid structure (TLS) is particularly important (12–14). Understanding the tumor microenvironment, especially TLS, will help boost ICI response rates (14). Only 40% of patients with HCC have TLS. TLS, which refers to the aggregation of lymphocytes in the tumor and at the edge of tumor invasion, has a function similar to lymph node and is an important part of the TME (15). In the future, ICI combined with TLS may be a key way to cure tumors.

Ferroptosis is a newly discovered process of apoptosis, which is closely related to iron metabolism (16). Studies show that ferroptosis can improve the immunotherapy response and inhibit tumor progression (17). ICI combined with ferroptosis-targeted therapy represents a new strategy to prolong the survival time of patients with HCC (18), and it has the potential to cure tumors.

Long non-coding RNAs (lncRNAs) does not have the function of coding protein, and the base length of genome is transcribed into noncoding RNAs, most of which have a great impact on the biological function of cells (19). Different lncRNAs play various roles in cell metabolism, such as promoting cancer growth, suppressing tumor growth, regulating immune responses, and enhancing the tumor infiltrating lymphocytes (20). For example, lncRNA CYTOR upregulates the expression of oncogene KIAA1522, which in turn affects the proliferation, apoptosis, and cell cycle of HCC cells (21).

The current problem of immunotherapy is that the response rate is low. In the face of this problem, we first need to find out the population with high sensitivity and then develop different methods to improve sensitivity for different groups, so as to achieve the effect of long-term survival or tumor cure. Therefore, we used multiple immune-related lncRNAs (irlncRNAs) from the Cancer Genome Atlas (TCGA) database to solve the problem. Our findings lay the foundation for the precise treatment of HCC so that more patients with HCC can benefit.

## Materials and Methods

### Data Download, Preparation, and Screening

HCC transcriptome data and corresponding clinical data were retrieved from the TCGA (https://portal.gdc.cancer.gov/), and immune-related gene sets were obtained from the gene set enrichment analysis (GSEA) (M19817 and M13664) (https://www.gsea-msigdb.org/). The irlncRNAs were identified by Spearman correlation test. Then, irlncRNAs associated with survival time were screened (p < 0.05). Patients with HCC whose survival time was <30 days and whose survival time was uncertain were excluded, leaving data from 343 patients for analysis, and the patients were randomly divided into a training set and a test set. In addition, 59 HCC transcriptome data (GSE40144) from the Gene Expression Omnibus (GEO) database was downloaded (https://www.ncbi.nlm.nih.gov/), as an external validation set.

### Construction of the Signature

LncRNAs associated with survival time were identified by the Cox proportional hazard regression model. The Akaike Information Criterion (AIC) was used to fit the optimal signature. The result with the lowest AIC value was selected to build the signature. RiskScore = expression(A) × cof(A) + expression(B) × cof(B) + …expression(n) × cof(n).

### Test of the Prediction Ability of the Signature

Kaplan–Meier survival curves were used to compare the survival times between the two groups, and multi-index receiver operating characteristic (ROC) curves were drawn to verify the ability of the signature to predict prognosis. Risk curves, survival status distribution maps, and heatmaps of the risk gene expression profiles were generated to further examine the reliability of the signature.

### Analysis of Clinicopathological Parameters

The pathological parameters were analyzed by Cox regression analysis. At the same time, the Wilcoxon signed-rank test was used to evaluate whether the riskScore was different among the groups of pathological parameters, and the chi-square test was used to analyze the relationship between clinicopathological parameters and the signature.

### Enrichment Analysis

Messenger RNAs (mRNAs) associated with risk genes were screened according to the principle of coexpression, and then STRING was used to analyze the interaction between coexpressed genes (https://string-db.org/). Finally, to understand the carcinogenic mechanism of disease risk genes, R software (4.0.1 version) was used for GO and KEGG pathway enrichment analyses. In addition, we used GSEA software to explore the role of risk genes in immunity (p < 0.05).

### Relationship Between the Signature and Clinical Treatment

To clarify the role of the signature in clinical treatment, the IC50 values of commonly used chemotherapeutic drugs were evaluated using high-throughput sequencing data of HCC in TCGA. The American Joint Committee on Cancer (AJCC) guidelines recommend the use of vincristine, cisplatin, and sorafenib in the treatment of HCC. The Wilcoxon signed-rank test was used to compare the differences between the two groups. Visualization of the results was performed using pRRophetic and ggplot2.

### Analysis of TILs

To determine whether the signature is associated with lymphocyte infiltration, we used the currently recognized online analysis websites TIMER (https://cistrome.shinyapps.io/timer/), CIBERSORT (http://cibersort.stanford.edu/), ssGSEA, and ImmuCellAI (http://bioinfo.life.hust.edu.cn) to calculate the degree of tumor lymphocyte infiltration in each sample. The correlation between the riskScore and TILs was tested by the Spearman test. The CIBERSORT results were used to evaluate the associations among TILs using Spearman analysis.

### Analysis of the Clinical Relevance of Immunotherapy

The immunophenoscore is calculated based on the expression of various important immune molecules in the TME, including major histocompatibility complex (MHC) molecules, immune regulatory factors, effector cells, and suppressor cells, which can well reflect the response rate of ICI. In addition, immune checkpoint gene expression is associated with the response to ICI; therefore, we analyzed the correlation among immune checkpoint genes and the IPS and the signature.

### Analysis of Genes Related to TLS Formation

The TLS helps to improve the immunotherapy response rate, so we explored whether genes related to TLS formation (CCL2, CCL3, CCL4, CCL5, CCL8, CCL18, CCL19, CCL21, CXCL9, CXCL10, CXCL11, CXCL13, and IL7) were associated with the signature.

### Analysis of Ferroptosis-Related Genes

Ferroptosis-related genes were collected from the GSEA database (M39768), and genes associated with survival (p < 0.05) were selected for further analysis.

### Statistical Analysis

All statistical analysis and plots were performed with R (R x64 4.0.3 version; https://www.r-project.org/). A p-value <0.05 was statistically significant. Difference analysis between two groups used Wilcoxon rank sum test. Relation analysis was identified by Spearman correlation analysis. Survival analysis was visualized by K–M curves and determined by log-rank test. Related R packages included “ggplot2,” “edgeR,” “ggpubr,” “survival,” “GSEABase,” and “GSVA.”

## Results

### Identification of Immune-Related LncRNAs Associated With Overall Survival Time

**Figure 1:**
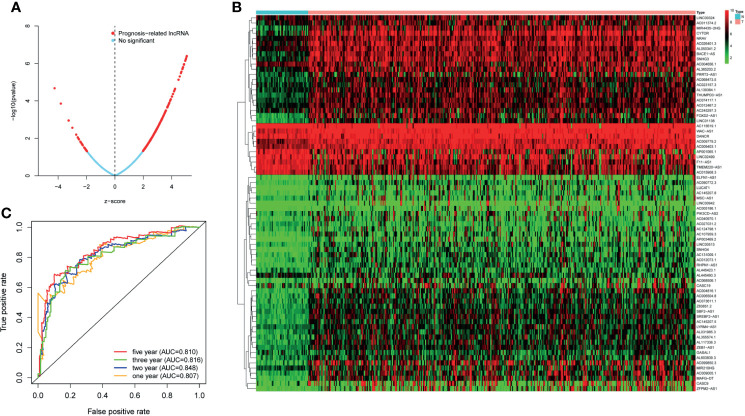
**(A)** The lncRNAs used to build the signature and the ability of the signature to predict the survival time. **(B)** LncRNAs associated with survival time. **(C)** The predictive ability of the signature for 1, 2, 3, and 5 years.

TCGA transcriptome data included 50 normal cases and 374 patients with HCC. A total of 14,121 noncoding RNAs and 19,628 mRNAs were obtained. A total of 1,327 irlncRNAs (R > 0.4, p < 0.001) were obtained by Spearman correlation analysis, of which 250 had significant effects on survival time (p < 0.05) (**Figure 1A**).

### Signature Identification

We selected 73 irlncRNAs (**Figure 1B**) associated with survival (p < 0.0002) for Cox proportional hazard regression model screening. Finally, nine differentially expressed genes (LINC00324, MSC-AS1, AC023157.3, AC009005.1 PRRT3-AS1, AC015908.3, AC145207.5, AL031985.3, and TMEM220-AS1) were selected to construct the signature. All patients were randomly divided into high-risk group (172 cases) and low-risk group (171 cases) according to the median value of riskScore calculated by R software.

### Verification of the Prediction Ability of the Signature

**Figure 2:**
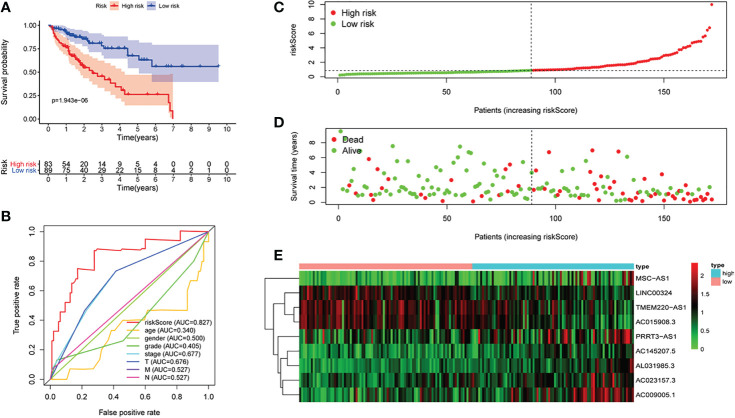
The signature of hepatocellular carcinoma (HCC) from the training set. **(A)** Kaplan–Meier survival curves, **(B)** multi-index ROC curves, **(C)** risk curves, **(D)** survival status distribution map, and **(E)** heatmap of the risk gene expression profiles.

**Figure 3:**
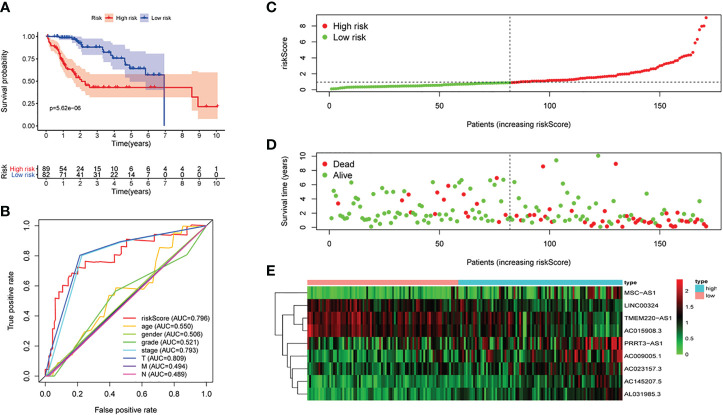
The signature of HCC from the testing set. **(A)** Kaplan–Meier survival curves, **(B)** multi-index ROC curves, **(C)** risk curves, **(D)** survival status distribution map, and **(E)** heatmap of the risk genes expression profiles.

**Figure 4:**
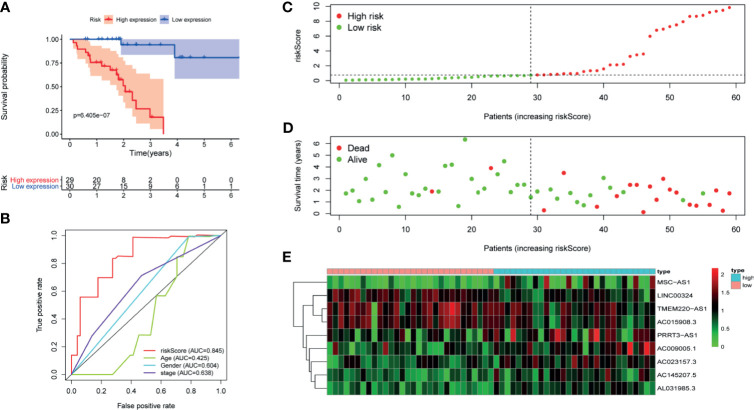
The signature of HCC from the external validation set. **(A)** Kaplan–Meier survival curves, **(B)** multi-index ROC curves, **(C)** risk curves, **(D)** survival status distribution map, and **(E)** heatmap of the risk genes expression profiles.

**Figure 5:**
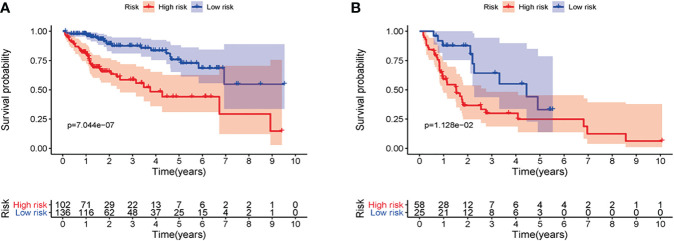
**(A)** Survival curve of early patients and **(B)** survival curve of advanced patients.

First of all, 343 patients were randomly put into a training set (83 high-risk cases and 89 low-risk cases) and a testing set (89 high-risk cases, 82 low-risk cases). Second, the training set (**Figures 2A–E**), the testing set (**Figures 3A–E**) and the external validation set (29 high-risk cases, 30 low-risk cases) (**Figures 4A–E**) were used to generate Kaplan–Meier survival curves, multi-index ROC curves, risk curves, survival status distribution maps, and the heatmaps of risk genes expression profiles. The results of the survival curves showed that patients in the low-risk group had significantly longer overalls survival time. The comparison of the area under the multi-index ROC curve revealed that the riskScore was better able to predict survival time than the traditional stage-by-stage method. Finally, to verify whether the signature was applicable to both the early and late stages of HCC, we drew the corresponding survival curves for both stages (**Figures 5A, B**). The results show that the high-risk group has significant difference with the low-risk group in survival time. In addition, areas under the ROC curve (AUCs) at 1 year (AUC = 0.807), 2 years (AUC = 0.848), 3 years (AUC = 0.816), and 5 years (AUC = 0.810) (**Figure 1C**) demonstrated the strong ability of the signature in predicting survival time. All of the above results showed that our signature has a high application value and reliability.

### Pathological Parameter Analysis

**Figure 6:**
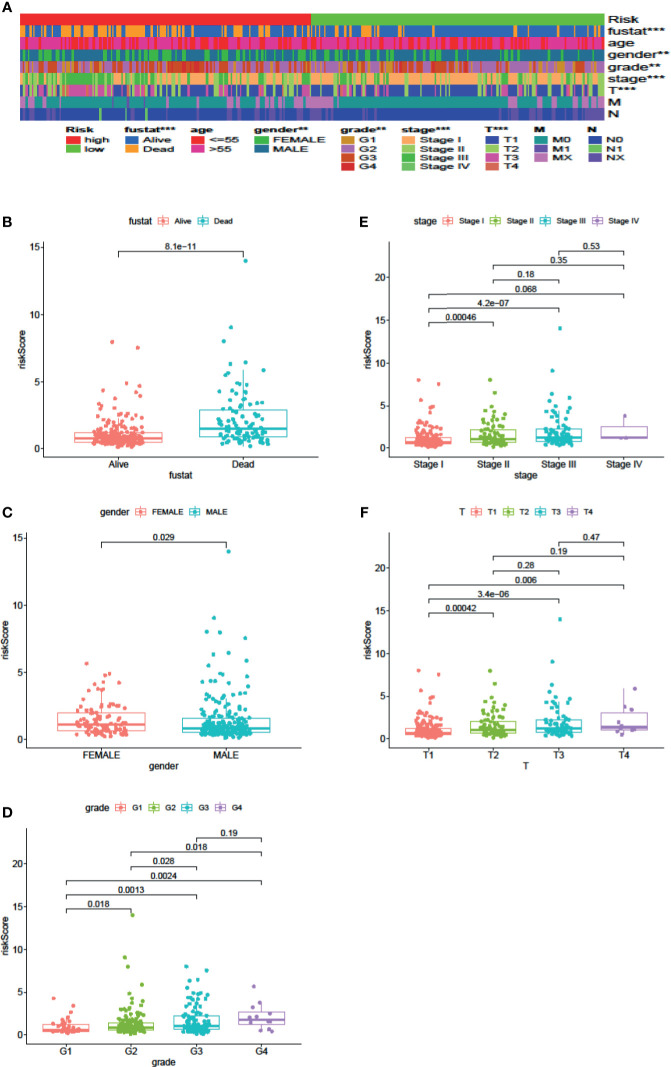
Clinical Evaluation by the Signature. A strip chart **(A)** along with the scatter diagram showed that survival status **(B)**, gender **(C)**, tumor grade **(D)**, clinical stage **(E)**, and T stage **(F)** were significantly associated with the riskScore (P <0.001 = ***, P <0.01 = **, and P <0.05 = *).

Cox regression analysis was used to analyze clinicopathological parameters (age, gender, survival status, grade, stage, T, N, M). The results are shown in **Table 1**. In addition, we used the chi-square test to verify the correlation between clinicopathological parameters and the riskScore (**Figure 6A**, ***p < 0.001, **p < 0.01, and *p < 0.05). Survival status, grade, stage, gender, and T stage were highly correlated to the riskScore, which was an independent prognostic factor. Then, to explore the difference in riskScores between different groups, we used the Wilcoxon signed-rank test. Results are presented in **Figures 6B–F**.

**Table 1:** Univariate and multivariate Cox regression analyses in each patient set.

Variables	Univariate analysis		Multivariate analysis	
	HR (95% CI)	p-value	HR (95% CI)	p-value
**Training set**				
Age	0.98 (0.95,1.01)	0.14		
Gender	1.01 (0.48,2.14)	0.98		
Grade	0.81 (0.50,1.32)	0.40		
Stage	1.55 (1.06,6.26)	0.02		
T	1.49 (1.04,2.14)	0.03		
M	6.66 (1.55,28.58)	0.01		
N	2.42 (0.33,17.90)	0.390		
riskScore	1.52 (1.30,1.78)	9.84E−08	1.61 (1.33,1.95)	1.16E−06
**Testing set**				
Age	1.01 (0.99,1.04)	0.35		
Gender	0.59 (0.31,1.14)	0.12		
Grade	1.20 (0.78,1.86)	0.40		
Stage	2.66 (1.83,3.88)	3.46E−07		
T	2.50 (1.79,3.50)	9.42E−08		
M	2.25 (0.31,16.59)	0.43		
N	1.37 (0.19,10.04)	0.76		
riskScore	1.36 (1.22,1.51)	4.51E−08	1.28 (1.14,1.45)	6.06E−05
**External validation set**				
Age	0.75 (0.29,1.93)	0.55		
Gender	1.07 (0.86,4.92)	0.07		
Stage	1.67 (1.15,4.25)	0.03	3.02 (1.02,8.90)	0.04
riskScore	4.51 (2.13,6.37)	0	5.53 (4.13,6.37)	0

### Enrichment Analysis

**Figure 7:**
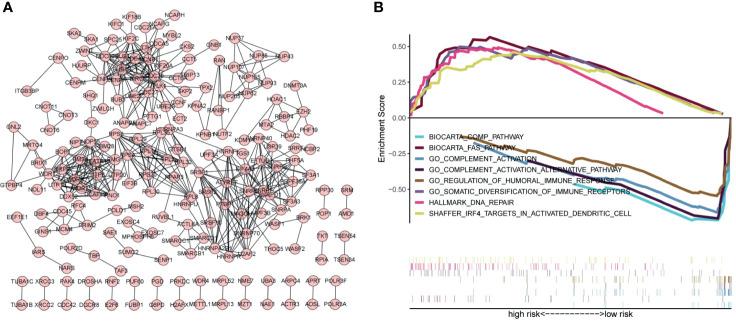
Protein–protein interaction networks and GSEA indicates the enrichment of significant pathways. **(A)** Protein–protein interaction networks. Each dot represents a protein molecule, and the connection between the dots means that the two molecules interact with each other. **(B)** Significant immune-related pathways enriched by GSEA.

**Figure 8:**
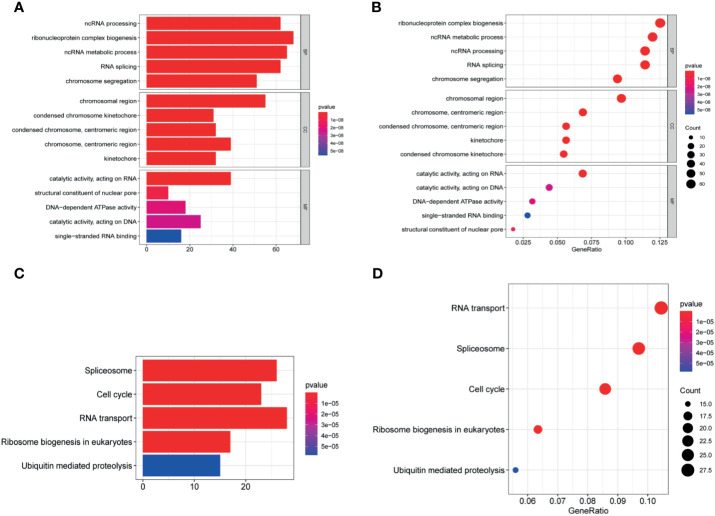
Enrichment analysis. **(A, B)** The significantly enriched GO terms and **(C, D)** KEGG pathways. The abscissa indicates the number and ratio of genes enriched in the pathway.

Exploring the biological functions of risk genes is helpful to understand their potential molecular mechanisms in tumorigenesis and development and can provide new targets for the treatment of HCC. According to the principle of coexpression, a total of 606 genes associated with risk genes (R > 0.4, p < 0.05) were identified, of which 604 were significantly differentially expressed. First, the significantly differentially expressed genes with a correlation coefficient >0.99 were selected for visualization (**Figure 7A**). The molecules CDC20, NOP56, SNRPG, SNRPF, BUB1B, and BMS1 were at the core of the protein interaction network. Second, to understand the carcinogenic mechanism of risk genes, coexpressed proteins were subjected to enrichment analysis. The results of Gene Ontology (GO) database enrichment analysis are shown in **Figures 8A, B**. The outcomes of the Kyoto Encyclopedia of Genes and Genomes (KEGG) database enrichment analysis are shown in **Figures 8C, D**. The results (**Figure 7B**) of GSEA suggested the importance of the risk genes for immune pathway regulation and showed that the high-risk group was in a state of immunosuppression.

### Application of the Signature in Clinical Treatment

**Figure 9:**
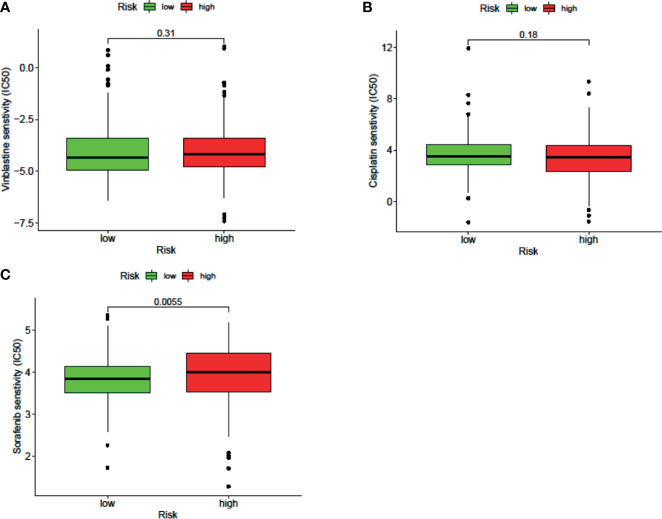
The signature can be used as a potential indicator to predict the sensitivity to sorafenib, and the sorafenib IC50 was higher in the high-risk group.

The results of the IC50 analysis of chemotherapeutic drugs are shown in **Figure 9**. The low-risk group showed a better response to sorafenib, but there was no significant difference in the response to vincristine or cisplatin.

### Analysis of TILs

**Figure 10:**
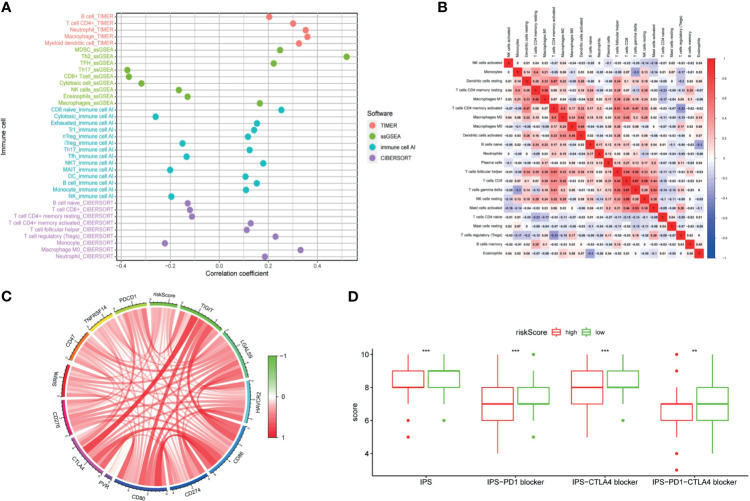
The signature was used to evaluate tumor-infiltrating lymphocytes, immunophenoscore (IPS), and immune checkpoint genes. **(A)** Spearman correlation analysis was used to calculate the correlation between the riskScore and the number of tumor-infiltrating lymphocytes. **(B)** Correlation heatmap of 22 tumor-infiltrating lymphocytes. **(C)** Analysis of the correlation between the riskScore and immune checkpoint genes. **(D)** The IPS, IPS-PD1 blocker, IPS–CTLA4 blocker, and IPS–PD1–CTLA4 blocker values are higher in the low risk group. (P <0.001 = *** and P <0.01 = **).

LncRNAs regulate immune-related genes that affect the TILs, which are terminal targets of ICI. Therefore, we explored whether there was a correlation between TILs and the riskScore. Results of CIBERSORT, TIMER, ImmuCellAI, and ssGSEA are shown in **Figure 10A**. Exhausted cells, macrophages, myeloid-derived suppressor cells (MDSCs), Treg cells, neutrophils, and eosinophils were positively correlated with the riskScore. Negative correlations were observed among Th17 cells, CD8+ T cells, cytotoxic T cells, NK cells, and mucosal-associated invariant T (MAIT) cells with the riskScore. In addition, the CIBERSORT analysis results showed a correlation among lymphocytes (**Figure 10B**). There was a weak to moderate correlation among various lymphocytes in the TME.

### Immunotherapy Analysis

The clinical application of ICI benefits many tumor patients, so we analyzed the relationships among immune checkpoint genes, immunophenoscore (IPS), and the signature. Our study found out that a riskScore was positively correlated with CD276, SIRPA, CD47, TNFRSF14, PDCD1, CTLA4, TIGIT, LGALS9, HAVCR2, CD86, CD274, CD80, and PVR expression (**Figure 10C**). The IPS analysis showed that the scores of IPS, IPS−PD1 blocker, IPS−CTLA4 blocker, and IPS−PD1−CTLA4 blocker scores were higher in the low-risk group (**Figure 10D**).

### Exploring Tertiary Lymphoid Structure-Related Genes

**Figure 11:**
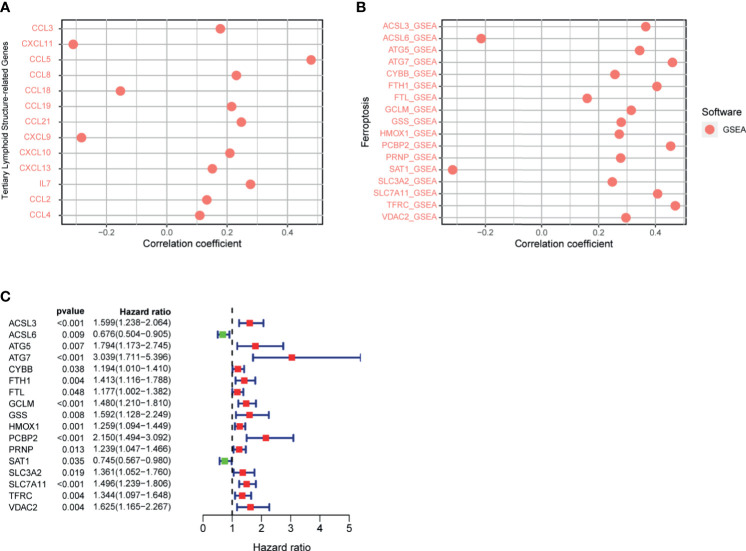
**(A)** Correlation between signature and genes related to tertiary lymphoid structure formation was calculated by Spearman. **(B)** Correlation between riskScore and ferroptosis-related genes was calculated by Spearman. **(C)** Ferroptosis-related gene univariate Cox regression analysis.

The TLS, an important part of the TME, has the ability to enhance the immune response to treatment. The genes related to TLS formation of CCL2, CCL3, CCL4, CCL5, CCL8, CCL19, CCL21, CXCL10, CXCL13, and IL7 were positively correlated with the riskScore (**Figure 11A**). Negative correlations were observed among CCL18, CXCL9, and CXCL11 with the riskScore. The data showed that the high risk group was genetically predisposed to form TLS, so the patients in the high-risk group were more likely to achieve a high response rate of immunotherapy through TLS in the future.

### Analysis of Ferroptosis-Related Genes

Previous studies have shown that ferroptosis increases the response to immunotherapy to enhance efficacy and inhibit tumor progression. Therefore, we analyzed the correlation between the signature and ferroptosis-related genes, which presented a positive correlation between the riskScore and the expression of ACSL3, ATG5, ATG7, CYBB, FTH1, FTL, GCLM, GSS, HMOX1, PCBP2, PRNP, SLC3A2, SLC7A11, TFRC, and VDAC2, respectively (**Figure 11B**). However, the expression of ACSL6 and SAT1 were negatively correlated with the riskScore, respectively. The above analysis revealed that the greater the riskScore, the weaker the degree of ferroptosis. The results of the COX univariate analysis of ferroptosis-related genes are shown in **Figure 11C**.

## Discussion

Increasing evidence demonstrated that lncRNAs seriously affect the occurrence, development, and metastasis of HCC and play an indispensable part in TME’s regulation process (22–24). The TME is not only related to the occurrence and development of cancers but also highly related to the response to ICI (25). At present, among the many treatment schemes for HCC, immunotherapy is the most popular choice and the most likely new therapy to make a considerable difference in patient outcomes. The future of HCC treatment is likely to involve immunotherapy combined with targeted therapy (26, 27). The postoperative recurrence and mortality rates of patients with HCC are high; the effective rate of ICI therapy is low. Our signature cannot only identify people who are sensitive to ICI but also select different methods to improve the response rate of ICI for different populations.

LINC00324 regulates FasL and PU.1 to promote the biological behavior proliferation, invasion, metastasis, and apoptosis of HCC stem cells (28). PRRT3-AS1 controls the mTOR signaling pathway to promote the invasion and metastasis and inhibits the autophagy and apoptosis of prostate cancer cells (29). MSC-AS1 induces PGK1 expression and accelerates the progression of HCC (30). In addition, MSC-AS1 has a similar role in gliomas (31), gastric cancer (32), renal clear cell carcinoma (33), and pancreatic cancer (34). TMEM220-AS1, AC015908.3, AC009005.1, and AL031985.3 have been reported in a signature predictive of overall survival. AC145207.5 and AC023157.3 are reported here for the first time. The survival curve was generated by the riskScore, and the results showed that the high-risk group has a significantly lower overall survival rate than the low-risk group. To validate the predictive ability of the signature, the areas under the ROC curve were calculated as AUC(1 year) = 0.807, AUC(2 year) = 0.848, AUC(3 year) = 0.816, and AUC(5 year) = 0.810, suggesting that the ability of our signature to predict overall survival was accurate and fines. Cox univariate and multivariate analyses revealed that the riskScore was an independent prognostic factor.

Better understanding of the TME will help develop new methods to modify the TME or treat HCC resulting in improving the response rate and efficacy of immunotherapy (35). At the same time, it will lay a solid foundation for the future implementation of immunotherapy combined targeted therapy. ICI rely on TILs against tumor cells. Therefore, we used CIBERSORT (36), ImmuCellAI (37), TIMER (38), and ssGSEA (39) to evaluate the relative number of TILs in each patient. As we expected, some of the lymphocytes showed differences between the two groups. Exhausted cells, macrophages, Treg cells, neutrophils, MDSCs, and eosinophils were more common in the high-risk group, while CD8+ T cells, cytotoxic T cells, natural killer (NK) cells, Th17 cells, and MAIT cells were more common in the low-risk group. Studies have found that the exhausted cells have a positive impact on the poor prognosis, and the completely exhausted CD8+ T cells do not respond to ICI (40, 41). Macrophages could increase the proliferation of HCC stem cells through the IL-6/STAT3 signaling pathway and then promote tumor growth (42). Treg cells regulate the expression of CTLA-4, PD-1, and TGF-β to directly bind with the corresponding receptors on target cells and inhibit the expression of the important cytokines interferon-gamma (IFN-γ), tumor necrosis factor-α (TNF-α), and IL-2 that affect T cells. In addition, tumor cells regulate chemokine receptor 28 (CCL28), thus recruiting Treg cells, enhancing the immune tolerance, and promoting the angiogenesis (43). The migration of neutrophils to tumors is mainly mediated by CXC chemokine binding to CXCR1 and CXCR2, which can promote tumor growth, invasion, angiogenesis, and metastasis (44). CD8+ T cells are positively correlated not only with good prognosis but also with the ICI response. Cytotoxic T cells specifically recognize the endogenous peptide MHC I complexes and kill tumor cells by expressing FasL or secreting TNF-α (43). The number of tumor-infiltrating NK cells positively correlated with the prognosis of HCC patients (45). Our results support the above conclusions. Considerable evidence shows that the TME is a whole, and all kinds of lymphocytes in the TME interact with each other (46). Our research results are consistent with this view. Therefore, the accuracy of analysis of the prognostic value of a certain type of cells alone is far lower than that of a combined multicell analysis. Studies that focus only on a specific kind of lymphocyte provide only a one-sided understanding of the TME and which cannot achieve a comprehensive, let alone a real, understanding of the TME. There were relatively few CD8+ T cells in the high-risk group, which may be one of reasons why more dendritic cells and shorter overall survival times were associated with the high-risk group. Moreover, our research found that there were more immunosuppressive cells and evading immune surveillance signals in the high-risk group, while more immune-enhancing cells and complement activation signals were found in the low-risk group. These observations revealed that the low-risk group is characterized by the immune activity and the inhibition of tumor progression. It also partially explained why the high-risk group is in a state of immunosuppression.

It has been reported that the IPS is a good predictor for the response of ICI (47), so we investigated the correlation between the IPS and the riskScore. IPS, IPS−PD1 blocker, IPS−CTLA4 blocker, and IPS–PD1–CTLA4 blocker were significantly higher in the low-risk group, indicating that the riskScore is the representative immunogenicity of TME in HCC. Higher gene expression level of immune checkpoint is one of the causes of an immunosuppressive state. According to a previous investigation, the anti-CTLA4 and anti-PD-1 treatment reactivated the antitumor immune response in the TME of HCC (48), and the expression of immune checkpoint genes was associated with the response to ICI (8). Our results demonstrated that the expression levels of CD276, SIRPA, CD47, TNFRSF14, PDCD1, CTLA4, TIGIT, LGALS9, HAVCR2, CD86, CD274, CD80, and PVR increased in the high risk group. The above results showed that the low-risk group of patients is more sensitive to treatment with ICI.

Only about 40% of patients with HCC have TLS within the tumor. Recent studies have reported that TLS, which have a similar function to the lymph node, enhance the sensitivity to ICI therapy (6). Therefore, the relationship between the molecules related to the formation of TLS and the signature was analyzed. Genes related to the formation of TLS, including CCL2, CCL3, CCL4, CCL5, CCL8, CCL19, CCL21, CXCL10, CXCL13, and IL7, were positively correlated with the riskScore, which indicated that patients in the high-risk group had a genetic advantage informing TLS. In the future, combining ICI with tertiary lymphoid structure-targeting therapy may improve the prognosis of patients; this gives patients who are not sensitive to ICI the opportunity to cure their tumors.

Ferroptosis enhances the effectiveness of ICI and inhibits the tumor progression (17). The expression of IFN-γ regulatory system X-c secreted by cytotoxic T cells enhances the sensitivity to ferroptosis (16). This suggests that ICI combined with ferroptosis inducers may become a new strategy for the treatment of cancer in the future. We analyzed 17 ferroptosis genes associated with the prognosis of HCC, of which 15 were positively correlated with the riskScore and 2 were negatively correlated. This suggests that the signature is fully capable of predicting tumor sensitivity to ferroptosis. In addition, in our study, the proportion of cytotoxic T cells in the low-risk group was significantly higher than that in the high-risk group. All this evidence suggests that patients in the low-risk group were not only more sensitive to ICI but also likely to be cured of their tumors by targeting ferroptosis in combination with immunotherapy.

It is important to recognize the shortcomings of the signature developed in this study: in clinical practice, screening out different treatments based on riskScore does not simply divide into high- and low-expression groups but requires more detailed subdivision. In future work, we need to collect more data to further verify the reliability of the findings.

## Data Availability Statement

The data presented in the study are deposited in the FigShare repository, accession link: https://figshare.com/s/93afe076e2ed13d32f4d.

## Author Contributions

WJS and YN conceived of and designed the study. YN, JHL, WLW, DNG and WJS performed the literature search, generated the figures and tables, and wrote the manuscript. JHL, WLW, XJL, TCZ, ZZM and YFW collected and analyzed the data, and critically reviewed the manuscript. WJS, YN, and DNG supervised the study and reviewed the manuscript. All authors contributed to the article and approved the submitted version.

## Funding

This study was supported by the National Natural Science Foundation of China (81672716, 81900571).

## Conflict of Interest

The authors declare that the research was conducted in the absence of any commercial or financial relationships that could be construed as a potential conflict of interest.

## Publisher’s Note

All claims expressed in this article are solely those of the authors and do not necessarily represent those of their affiliated organizations, or those of the publisher, the editors and the reviewers. Any product that may be evaluated in this article, or claim that may be made by its manufacturer, is not guaranteed or endorsed by the publisher.
